# Rare subgroup of A phenotype—a case series

**DOI:** 10.2478/abm-2026-0023

**Published:** 2026-06-30

**Authors:** Nur Ilyia Syazwani Saidin, Nurul Anis Che Anuar, Mohd Nazri Hassan, Noor Haslina Mohd Noor, Zefarina Zulkafli, Hisham Atan Edinur

**Affiliations:** Hematology Department, School of Medical Sciences, Health Campus, Universiti Sains Malaysia, 16150 Kubang Kerian,Kelantan, Malaysia; School of Health Sciences, Health Campus, Universiti Sains Malaysia, 16150 Kubang Kerian,Kelantan, Malaysia; Hospital Pakar Universiti Sains Malaysia, 16150 Kubang Kerian,Kelantan, Malaysia

**Keywords:** A phenotype, ABO discrepancies, ABO incompatibility, subgroup A, weak agglutination

## Abstract

Testing for ABO incompatibility between a potential blood donor and recipient is the cornerstone of pretransfusion testing. Occasionally, weak agglutination reactions occur due to low expression of A and B antigens on the surface of red blood cells. This can lead to inconsistencies in blood group typing, as seen in a case where a patient’s forward and reverse typing showed discrepancies. Following a comprehensive analytical assessment, the patient was identified as having a distinctive variant of blood group A, belonging to the less common subgroups—A_3_, A_end_, A_x_, A_m_, A_y_, and A_el_. Herein, we present 3 case series of ABO subgroups identified through extended ABO blood grouping procedures.

The ABO blood group system was first described by Karl Landsteiner in 1900 and serves as the fundamental basis for compatibility testing in transfusion medicine. The A gene, located on chromosome 9, encodes the transferase enzyme N-Acetylgalactosaminyltransferase. This enzyme facilitates the transfer of immunodominant sugar N-acetylgalactosamine onto the H antigen, resulting in the formation of the A antigen [1].

The ABO blood group system is the most clinically significant in transfusion medicine, and discrepancies may occur when forward (red cell) and reverse (plasma/serum) typing results do not align. These discrepancies can result from weak or variant antigen expressions, technical errors, unexpected antibodies, rouleaux formation, or underlying disease. They are observed in both donors and patients and must be carefully resolved to ensure safe transfusion. Desai et al. [2] highlighted the importance of systematic serological resolution, while Arumugam et al. [3] showed that stepwise testing, including extended grouping and secretor studies, effectively resolved such cases.

Laboratory workup of ABO discrepancies involves repeating the test to exclude technical error, reviewing patient history (e.g., transfusion, transplantation, age, and disease), using additional serological techniques (e.g., testing with anti-A_1_ lectin, adsorption-elution studies, and secretor status determination), and, when needed, molecular genotyping to identify underlying ABO variants. Accurate resolution is essential to avoid misclassification and ensure transfusion safety [4,5,6].

The group A phenotypes are classified into 2 major subgroups: A_1_ and A_2_. Additional subgroups include A_3_, A_x_, A_m_, A_end,_ A_el_, A_m_, and A_y_. These A subgroups have different abilities to convert H antigen to the A antigen. The A_1_ subgroups have the greatest capacity for conversion and express the largest number of antigenic sites on the red blood cell surface, with approximately 2 million A antigens. In contrast, the A_2_ phenotypes display around 500,000 A antigens on adult red cells, while the rarer A subgroups express significantly fewer A antigens [7]. The decreased number of A antigen sites per red cell results in weak or absent agglutination when tested with commercial anti-A reagents. Other subgroups, such as A_3_, may show a mixed-field reaction with anti-A. In addition, anti-A_1_ alloantibody can be detected in the serum of individuals with A2, A3, Ax_,_ and Ael. phenotypes [7].

The rare A subgroups arise from mutations in the ABO gene that reduce or abolish A transferase activity, resulting in variable expression of the A antigen. A_3_ typically shows partial loss of enzyme activity with mixed-field agglutination due to uneven A antigen expression; A_x_ results from more severe mutations, producing very weak reactions with anti-A but strong with anti-AB, often resembling group O; A_m_ and A_y_ have minimal to absent A antigen on red cells, but A_m_ individuals (if secretors) can still produce soluble A substance in secretions, whereas A_y_ shows absence in both cells and secretions; and A_en_ variants, due to mutations at the 3′ end of the gene, show very weak red cell expression but may retain normal secretion. Thus, the genotypic variations dictate the degree of enzyme activity, and phenotypically this ranges from weak, mixed, or absent red cell A antigen expression to differences in secretory status [4, 6].

A recent study found a prevalence of 99% for the A1 subgroup and 1% for the A2 subgroup. This study also identified the absence of the 1,061°C deletion in A_2_ blood groups [8]. Other A subgroups are much more infrequent, constituting less than 1% frequency due to the inheritance of rare alleles at the ABO locus [7]. Here, we present a case series of this unusual A subgroup, distinct from A_1_ phenotypes, with their implications in the transfusion decisions. Written informed consents were obtained from the patients for publication of the clinical details.

## Case 1

A 29-year-old female, gravida 3 para 2, at 37 weeks of gestation, presented with spontaneous vaginal bleeding. The ultrasound revealed bleeding from placenta previa type 3 anterior. Upon admission, she had mild anemia, with a hemoglobin level of 10.8 g/dL. In anticipation of a need for a blood transfusion, a blood sample was obtained for pretransfusion testing. However, an issue arose as the blood group determination became inconclusive due to an ABO typing discrepancy. The forward blood grouping yielded a reaction indicative of group O, while the reverse blood grouping showed group A due to the presence of anti-B antibodies. She underwent an elective lower segment cesarean section (LSCS) and the procedure was carried out without any complications, and there was no requirement for a blood transfusion.

## Case 2

A 41-year-old female, with gravida 5 para 4, at 36 weeks of gestation, was admitted for elective LSCS. She had underlying diabetes mellitus and hypertension with history of 2 previous LSCS for the delivery of a macrosomic baby. A blood sample was sent for pretransfusion testing. ABO typing discrepancies were detected in her blood grouping. There was a weak reaction observed with anti-A (2+), and a strong reaction with B cells (4+). Extended ABO blood grouping was done, suggesting the possibility of an A subgroup. She underwent her third LSCS along with a hysterectomy due to placenta accreta. The procedure was complicated by primary postpartum hemorrhage. She received 2 units of group O packed red cells.

## Case 3

A 25-year-old female was involved in a motor vehicle accident and sustained a left-sided femur fracture with an abrasion wound on her forehead. Upon admission, her hemoglobin levels were 11 g/dL. She was planned for the implantation of an interlocking nail for the left femur fracture. A blood sample for pretransfusion testing was sent to the blood bank. There were ABO typing discrepancies detected during blood grouping. Extended ABO blood grouping was done. The presence of a mixed field reaction with anti-A and a strong reaction with B cells (4+) raised suspicion of an A subgroup.

The results of extended blood grouping for the 3 cases were summarized in **Table 1**, which categorized all 3 cases under the A subgroup. This is done by the addition of Anti-H and Anti-A_1_ lectin for forward grouping and secretor study.

**Table 1.** The extended ABO blood grouping

	**Forward grouping**						**Reverse grouping**				**Secretor status**	**Possible Subgroup**
	
	**Anti-A**	**Anti-B**	**Anti-A, B**	**Anti-D**	**Anti-H**	**Anti-A1**	**A1 Cells**	**A2 Cells**	**B Cells**	**O Cells**		
**Lectin**												
Case 1	0	0	0	4+	4+	0	0	0	4+	0	ND	A_el_
Case 2	2+	0	1+	4+	3+	0	0	0	4+	0	Present of H antigen in saliva	A_x_
Case 3	++MF	0	++MF	4+	4+	0	0	0	4+	0	ND	A_3_

MF, mixed field; ND, not done.

Case 1 suggests A_el_ subgroup in the patient. Although the Parabombay A phenotype was initially considered, the strong reaction with Anti-H exclude this possibility. The saliva secretor test was not performed due to the lack of a sample to further confirm the A_el_ phenotypes.

In case 2, the patient may possibly have A_x_ phenotypes. The saliva secretor test demonstrated the presence of only H substances. The initial reaction observed in this case could also be seen in the A_2_ subgroup; however, A_2_ subgroup individuals typically show A and H substances in their saliva.

For case 3, the patient’s phenotype might align with A_3_. Mixed field reactions in the forward grouping are commonly observed in the A_3_ subgroup. Although A and H soluble antigens are anticipated in the saliva secretor study, the unavailability of samples precluded further confirmation of the phenotype.

## Discussion

ABO blood group antigens continue to hold paramount significance in transfusion medicine due to their highest immunogenicity compared to other blood group antigens. The primary cause of fatalities resulting from blood transfusions is attributed to clerical errors that lead to the inadvertent transfusion of incompatible ABO blood types. The ABO blood group antigens have demonstrated significance over the course of human evolution, as evidenced by the varying frequencies of different ABO blood types across diverse populations [9].

A large study from China reported the prevalence and distribution of weak ABO subgroups, highlighting the variability of serological characteristics in the Chinese population [10]. Similarly, in Malaysia, extended blood group profiling across Malays, Chinese, and Indians showed the presence of rare ABO subgroup alleles, demonstrating ethnic variation within Southeast Asia [11]. These findings highlight the importance of regional data in understanding ABO subgroup diversity and its relevance to transfusion safety.

About 20% of individuals exhibiting the A antigen are attributed to the A_2_ subtype, subsequently forming either the A_2_ or A_2_B subgroup. Conversely, the remaining majority is encompassed by the A_1_ subtype, leading to the constitution of either the A_1_ or A_1_B subgroup [12].

Weak subgroups of A may present practical problems. A_x_ donor may mistype as group O and transfused to a group O patient, causing rapid intravascular hemolysis [1].

Extended ABO typing, secretor studies, adsorption-elution test, and molecular testing can be utilized to subdivide an A individual into A_3_, A_x_, A_end_ etc (**Table 2**).

**Table 2.** Characteristics of weak ABO phenoptypes

**Phenotypes**	**Forward grouping**			**Reverse grouping**			**Other substances present in saliva**
	
	**Anti A**	**Anti B**	**Anti H**	**Anti A**	**Anti B**	**Anti A1**	
A_3_	++mf	0	3+	no	yes	sometimes	A, H
A_X_	weak/0	0	4+	0/weak	yes	almost always	H
A_end_	weak mf	0	4+	no	yes	sometimes	H
A_m_	0/weak	0	4+	no	yes	no	A, H
A_y_	0	0	4+	no	yes	no	A, H
A_el_	0	0	4+	some	yes	yes	H

Adapted from [1]. mf, mixed field agglutination; 0, negative.

For definite classification, molecular techniques are available to characterize the genotype if necessary. At present, more than 200 ABO allele genes have been found, formed by mutations, such as base insertion, omission, point mutation, gene recombination, and exchange [13]. A mutation (c.745 C > T) in exon 7 of the ABO blood group gene resulted in low activity of α-1, 3-N-acetyl-galactosaminyl transferase, producing A_3_ phenotype [13].

The A_3_ blood group is very heterogeneous at the molecular level. Suxuki [14] reported that samples of 4 unrelated A_3_ blood donors showed heterozygosity for the G261 deletion. In the A_3_ allele, mutation and deletion in C467T, 1,060°C and G829A were found. One blood donor presented the T646A and the G829A mutations in homozygosity [14].

The A_x_ phenotype has been associated with many different A variant alleles. Nair et al. reported that the molecular genotype of A_x_ was found to be Aw06/O13 [15].

The occurrence of hybrid alleles can also explain hitherto abnormal inheritance in some pedigrees. The detection of hybrid alleles has been made possible by the presence of numerous polymorphisms in the various ABO alleles [16].

A new genotyping approach has been developed and evaluated that can correctly identify ABO alleles, including non-deletional null alleles, subgroups, and hybrids resulting from recombinational crossing-over events between exons 6 and 7. This approach is clinically applicable and decreases the risk for erroneous ABO phenotype prediction [17].

The main limitation of this case series is the incomplete laboratory workup for some patients. Secretor status testing and molecular studies, which are important for definitive differentiation of A subgroups, could not be performed due to the unavailability of samples, loss to follow-up, and resource constraints.

## Conclusion

Additional special procedures, such as molecular testing for mutations or serum glycosyltransferase studies for detecting the A enzyme can be performed for differentiation of weak subgroups. In our patients, molecular testing was not done due to the unavailability of the test in our institute. In those cases, unmatched universal donor products like O-negative/positive red blood cells are transfused simultaneously with the workup of the discrepancy. It’s important to ensure that any discrepancies in test results are resolved and well-documented before reporting the patient’s blood group [18].
